# The long-term safety and effectiveness of the loop technique in left ventricular lead dislocation

**DOI:** 10.3389/fcvm.2023.1154125

**Published:** 2023-09-04

**Authors:** Mengya Dong, Chenyuan Liang, Gong Cheng

**Affiliations:** Department of Cardiovascular Medicine, Shaanxi Provincial People’s Hospital, Xi’an, China

**Keywords:** heart failure, cardiac resynchronization therapy, loop technique, left ventricular lead dislocation, new technique

## Abstract

**Objectives:**

Cardiac resynchronization therapy (CRT) is a well-established method that improves the clinical symptoms and long-term prognosis of specific heart failure (HF) patients by restoring systolic synchronicity and enhancing myocardial function. However, the high rate of intraoperative and postoperative left ventricular (LV) lead dislocation limits its application to a great extent. The aim of this study was to demonstrate the long-term safety and effectiveness of a new approach named the loop technique for patients who experience repeated intraoperative transvenous LV lead dislocations during CRT.

**Methods:**

The current study was a single-centre, prospective, nonrandomized controlled trial. Forty-four HF patients who underwent CRT were included. All patients were followed to death or 3 years.

**Results:**

Among 44 HF patients, 36 underwent the traditional operation, and 8 underwent the loop technique due to repeated intraoperative LV lead dislocations. Intergroup comparison revealed no significant differences between the two groups with respect to most preoperative indices, intraoperative pacing and sensing parameters. At the end of the 3-year follow-up, 4 (11.1%) patients in the traditional operation group and 2 (25.0%) patients in the loop technique group had died. There was no significant difference in the mortality rate (*P* = 0.30). No complications related to this new technique were observed, such as intracoronary thrombosis, infection or dislocation. Intergroup comparison showed no significant difference in the New York Heart Association (NYHA) class, echocardiography indices, N-terminal pro brain natriuretic peptide (NT-proBNP) level or pacemaker programming parameters.

**Conclusions:**

The loop technique is a safe and effective alternative method for patients who experience repeated intraoperative transvenous LV lead dislocations during CRT.

## 1. Introduction

Heart failure (HF), a clinical syndrome and an end stage of various cardiovascular diseases (CVDs), results from a structural or functional abnormality in ventricular filling or ejection of blood. Despite better management of CVD, the overall incidence and prevalence of HF are increasing (1, 2). Therefore, optimized diagnostic and therapeutic algorithms are still urgently needed.

Cardiac resynchronization therapy (CRT) is a well-established method that improves the clinical symptoms and long-term prognosis of specific HF patients by restoring systolic synchronicity and enhancing myocardial function (3). However, several perioperative and postoperative complications, especially left ventricular (LV) lead dislocation, hinder its widespread application (4). The failure rate of LV lead implantation could be as high as 10% to 15% (5). Although some techniques have been applied to improve this situation, the occurrence of unsuccessful lead positioning and/or dislocation is still 5%–10% (6). Therefore, new methods to solve this problem is significant for CRT.

In the present study, we first reported the safety and effectiveness of a brand-new technique named the loop technique, which we specifically devised for patients who experience repeated intraoperative transvenous LV lead dislocations during CRT.

## 2. Methods

### 2.1. Study population

The current study was a single-centre, prospective, nonrandomized controlled trial. From January 2013 to June 2019, a total of 56 patients who were scheduled for CRT were screened for this study at the Cardiology Department of Shaanxi Provincial People's Hospital. The inclusion criteria were as follows: (1) patients aged between 18 and 80; (2) patients who were able to understand the purpose of the study and who signed the informed consent form voluntarily; (3) patients who were diagnosed with New York Heart Association (NYHA) class III or higher advanced HF despite optimal medical therapy; (4) patients who had a prolonged QRS duration > 130 ms; (5) patients who met the criteria for CRT and who planned to undergo CRT (7); (6) patients who underwent echocardiography and had a left ventricular ejection fraction ≤ 35%; and (7) patients with a clear and readable angiography image.

The exclusion criteria were as follows: (1) women who were pregnant, breastfeeding, or planning to become pregnant; (2) patients with a history of myocardial infarction in the last 30 days prior to CRT; (3) those who had previously undergone coronary artery bypass surgery or who had been implanted with a pacemaker, an implantable cardioverter defibrillator, or an artificial heart valve; (4) patients with an allergy to contrast media; (5) patients with liver or renal dysfunctions; (6) patients who had not undergone preoperative echocardiography; (7) patients with an unqualified angiography image; (8) patients with malignant tumours; and (9) patients with other circumstances that were not suitable for participating in the experiment.

**Figure 1:**
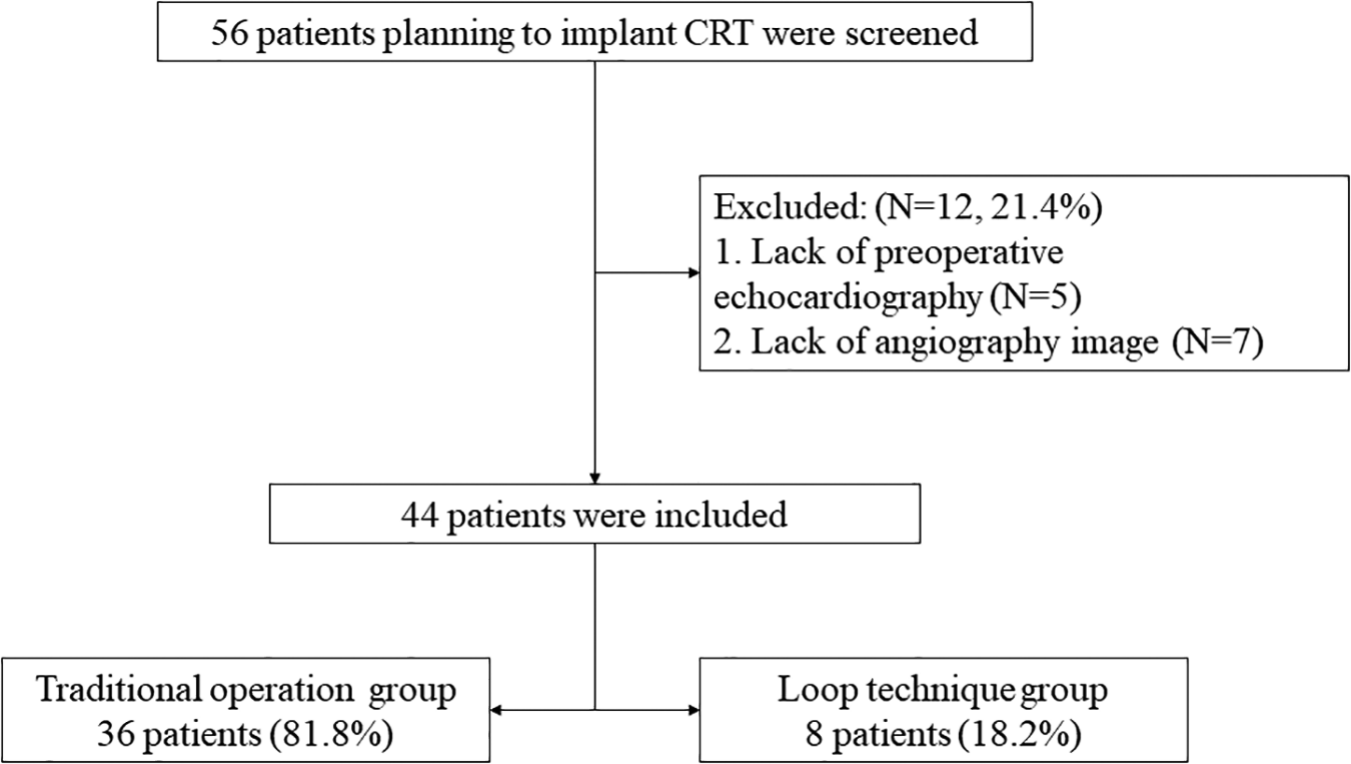
Cohort selection flow diagram.

The flowchart of the analysis is presented in Figure 1, and 44 patients participated in the study. This study complied with the Declaration of Helsinki (as revised in 2013) and was approved by the Ethics Committee of the Shaanxi Provincial People's Hospital, Xi'an, Shaanxi, China (No. SPPH-LLBG-12-3.2). Written informed consent was obtained from all study participants.

### 2.2. Data collection

The collected data included patients' demographics, medical histories, results of laboratory testing and echocardiography images at admission.

### 2.3. Loop technique in CRT

**Figure 2:**
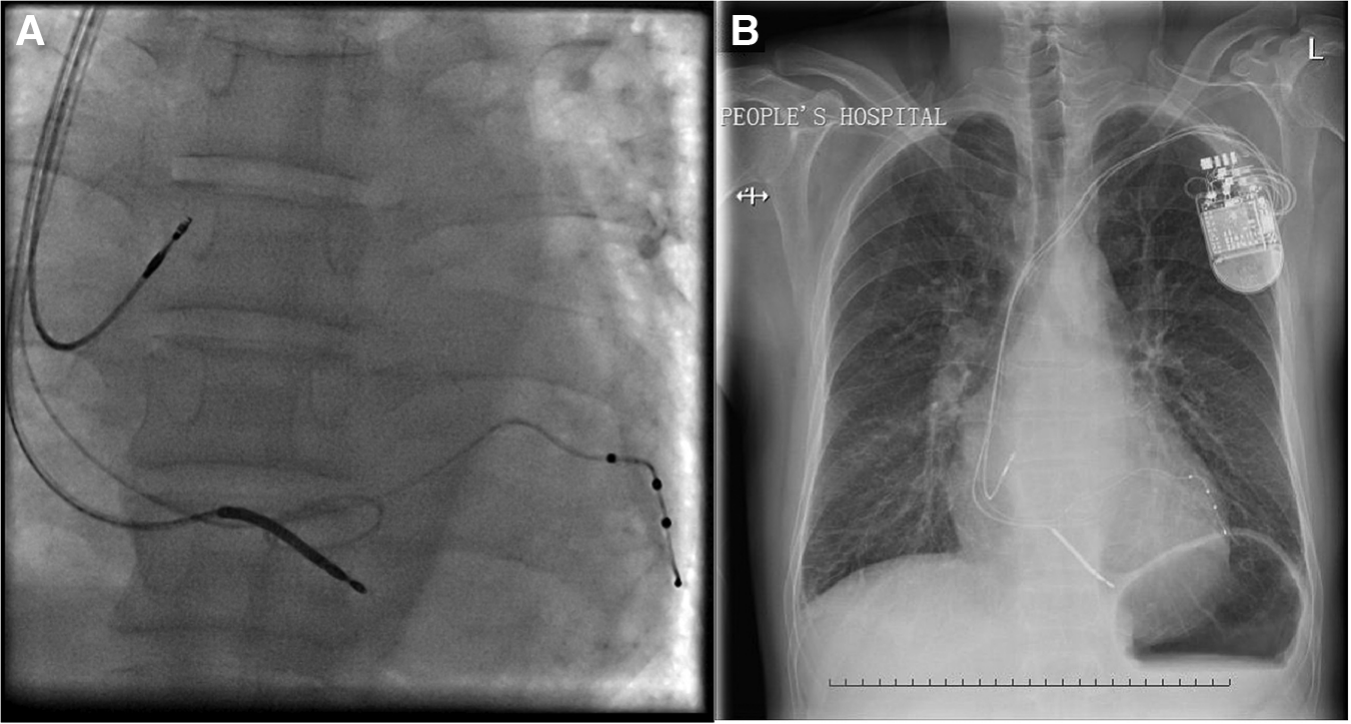
(**A**) Final position of the left ventricular lead by the loop technique from right anterior oblique projection. (**B**) Chest x-ray image of the position of the left ventricular lead by the loop technique at the 3-year follow-up.

The loop technique in CRT has been published previously (8). Basically, the loop technique was performed to position the left ventricular lead in cases of repeated left ventricular lead dislocations (≥2 dislocations), and at least two different manufacturers' lead configurations were tried. The left ventricular lead was looped through a vessel adjacent to the target vessel. The parameters of CRT were recorded (Figure 2A).

### 2.4. Follow-up

The endpoint was all-cause mortality. Patients were followed up by face-to-face interviews by well-trained cardiologists. The end of follow-up was the date of the endpoint occurrence or 3 years after the operation. In addition, at the 3-year follow-up, chest x-ray imaging, echocardiography, laboratory testing and pacemaker programming were repeated.

### 2.5. Statistical analysis

Continuous variables are presented as the mean ± standard deviation (SD) or median [lower quartile, upper quartile]. The Kolmogorov‒Smirnov test was used to assess the normality of continuous variable distributions. Student's t test and the Mann‒Whitney U test were used for the comparison of continuous variables as appropriate. Categorical variables are presented as frequencies (percentages). The *χ*^2^ test was used to analyse the differences between categorical variables. All computations were performed with SPSS software v22.0 (SPSS Inc., Chicago, Ill., USA). A statistically significant difference was defined at *P* < 0.05 using a two-tailed test.

## 3. Results

### 3.1. Clinical characteristics of the study population

The 44 patients were divided into 2 groups according to the type of CRT: the traditional operation group (*n* = 36, 81.8) and the loop technique group (*n* = 8, 18.2). The main preoperative characteristics of these 2 groups are shown in Table 1. The N-terminal pro-brain natriuretic peptide (NT-proBNP) level was higher in the traditional operation group than in the loop technique group (*P* = 0.03). Intergroup comparison revealed no significant differences between the two groups with respect to most preoperative indices.

**Table 1:** Preoperative characteristics of patients with heart failure according to operations of CRT implantation.

Variable	Traditional operation group	Loop technique group	*P* value
	*n* = 36	*n* = 8	
Males (%)	22 (61.1)	4 (50.0)	0.68
Age (years)	61.8 ± 8.8	59.7 ± 5.6	0.67
Underlying heart disease			
Dilated cardiomyopathy	24 (66.7)	6 (75.0)	0.66
Coronary artery disease	6 (16.7)	2 (25.0)	
Both	6 (16.7)	0 (0.0)	
NYHA			
III	30 (83.3)	6 (75.0)	0.70
IV	6 (16.7)	2 (25.0)	
Echocardiography indexes			
LVEF (%)	30.6 ± 9.1	31.3 ± 10.3	0.90
LVESD (mm)	63.8 ± 11.0	65.3 ± 18.3	0.83
LVEDD (mm)	72.6 ± 10.6	73.3 ± 17.0	0.92
NT-proBNP (pg/ml)	2453.0 [976.3, 3573.0]	575.0 [218.0, 865.3]	0.03

In the traditional operation group, 28 (77.8%) underwent the procedure with ST. JUDE medical/1458/86 cm/7 Fr, and others underwent the operation with Medtronic/4195/78 cm/8 Fr. In the loop technique group, the numbers were 5 and 3, respectively, and there was no significant difference (*P* = 0.37). Intraoperative pacing and sensing parameters were also recorded. In the traditional operation group, the threshold and impedance of the left ventricular lead were 0.95 [0.75, 1.50] and 771.50 [561.75, 931.75], respectively. In the loop technique group, the threshold and impedance were 1.43 [1.00, 3.09] and 826.50 [705.00, 908.25], respectively. There were no significant differences in these parameters between the two groups (*P* = 0.14 and *P* = 0.84).

### 3.2. Follow-up and long-term prognosis

During the follow-up, 4 (11.1%) patients in the traditional operation group and 2 (25.0%) patients in the loop technique group died. There was no significant difference in the mortality rate (*P* = 0.30). All the leads worked properly in all patients. In addition, in the loop technique group, there were no complications related to this new technique, such as intracoronary thrombosis and infection. The remaining 38 patients were followed for three years. At the end of the follow-up, the locations of the electrode and leads were checked by chest x-ray imaging, and no lead dislocation occurred (Figure 2B). As shown in Table 2, the intergroup comparison showed no significant differences with respect to NYHA class, echocardiography indices and NT-proBNP level.

**Table 2:** Postoperative characteristics of patients with heart failure according to operations of CRT implantation.

Variable	Traditional operation group	Loop technique group	*P* value
	*n* = 32	*n* = 6	
NYHA			
II	10 (31.3)	2 (33.3)	0.60
III	14 (43.7)	4 (66.7)	
IV	8 (25.0)	0 (0.0)	
Echocardiography indexes			
LVEF (%)	35.1 ± 11.1	42.0 ± 18.2	0.38
LVESD (mm)	58.9 ± 17.0	54.0 ± 27.9	0.68
LVEDD (mm)	69.7 ± 15.9	62.3 ± 25.1	0.51
NT-proBNP (pg/ml)	925.5 [688.0, 1225.0]	521.0 [373.0, 0.0]	0.29

Postoperative pacing and sensing parameters were also recorded through pacemaker programming. In the traditional operation group, the threshold and impedance of the left ventricular lead were 1.05 [0.81, 1.50] and 783.00 [683.75, 935.25], respectively. In the loop technique group, the threshold and impedance were 1.87 [1.05, 3.50] and 903.00 [790.00, 910.00], respectively. There were still no significant differences in these parameters between the two groups (*P* = 0.14 and *P* = 0.63).

## 4. Discussion

Although CRT uses advanced technology to ameliorate the symptoms and prolong the survival of some HF patients, the high incidence of LV lead dislocation still affects its clinical effectiveness. Our team first developed the loop technique, and the current study reported that the loop technique not only solved LV lead dislocation but also achieved an ideal therapeutic effect. To the best of our knowledge, our study is the first analysis of the long-term safety and effectiveness of the loop technique for patients who experience repeated intraoperative transvenous LV lead dislocations during CRT.

Clinical scientists first attempted to reduce the dislocation rate by changing surgical incisions. Initially, epicardial pacing lead implantation was the most frequently used alternative for patients with transvenous intraoperative and/or postoperative LV lead dislocation. The following studies proposed that transapical endocardial CRT was a better choice because of its shorter procedure time and decreased postoperative burden. Kassai et al. first proposed transapical implantation of the endocardial LV lead as a feasible approach to decrease the dislocation rate (9, 10). Ten HF patients were implanted with LV leads through this method, and 1 suffered from lead dislocation and another encountered pocket infection. Further studies from this team and other researchers also draw similar conclusions (11, 12). The latest research showed that transseptal endocardial LV lead implantation was another effective method; however, the approach was related to a substantial thromboembolic risk (13).

Subsequently, the renewal technologies of components of CRT were also used to solve the high rate of LV lead dislocations. Recently, a quadripolar transvenous lead for CRT could reduce the dislocation rate and share similar curative effects with the conventional implant method (14). Luedorff et al. reported that the use of an active fixation LV lead also improved the success rate of CRT (15).

In addition, clinicians have attempted to use stents to anchor the LV lead (16–18). They implanted a stent in a coronary sinus branch in 312 patients and conducted long-term follow-up observation. The results showed that it was probably an effective and safe procedure to prevent and treat lead dislocation, although a few patients had an increased LV pacing threshold, phrenic nerve stimulation or infection.

In addition, previous researchers tried to retain a guidewire to stabilize the lead in cases of repeated intraoperative dislocations, which is similar to our results (19). However, the coiled guidewire might fracture the lead due to prolonged friction.

Recently, physiological pacing options, such as His bundle pacing (HBP) and left bundle branch area pacing (LBBAP), have been shown to be superior to conventional biventricular pacing (BVP) modalities when LV lead implantation fails (20–22). HBP is defined as capture of the atrioventricular bundle by directly activating all of its fibres (23). A few studies showed that HBP was not inferior to BVP in shortening the QRS duration or improving the LVEF (24, 25). However, other data revealed that HBP could not improve patient prognosis and showed higher pacing thresholds (26, 27). LBBAP is defined as capture of the predivisional LBB to reach the same ends and is associated with a higher success rate and a lower complication rate than HBP (23, 28–31). However, some complications with LBBAP are still noteworthy, including septal perforation, right bundle branch block and complete heart block (23).

Beyond that, some scientists suggested that a systematic approach to every step of the implantation process could upgrade the success of CRT implantation (32). It was also an alternative solution to take full advantage of the existing anatomical structure to solve the failure of LV lead implantation (21).

Compared with the above strategies, the loop technology we proposed has the following outstanding advantages. First, the dislocation rate was extremely low. No LV lead dislocation was observed in the loop technology group. Second, the loop technique was a minimally invasive approach compared to the traditional procedure and did not require extra skin incisions. Therefore, patients suffered less pain, and the infection rate was lower. Third, the loop technology only used original devices without changing electrodes or leads or adding stents; thus, this procedure does not increase costs, thus decreasing the economic burden for patients.

The main limitation of our current study is the small sample size. Although the therapeutic effects were satisfying, no relevant complications were observed in the 8 patients who underwent the loop technique. The results still need to be confirmed in a multicentre study with a large sample size.

In conclusion, the loop technique is a safe and effective alternative method for patients who experience repeated intraoperative transvenous LV lead dislocations during CRT.

## Data Availability

The datasets analysed during the current study are available from the corresponding author on reasonable request.
